# Stress-related transcriptomic changes associated with GFP transgene expression and active transgene silencing in plants

**DOI:** 10.1038/s41598-024-63527-5

**Published:** 2024-06-10

**Authors:** Paraskevi Kallemi, Frederic Verret, Christos Andronis, Nikolaos Ioannidis, Nikolaos Glampedakis, Kiriakos Kotzabasis, Kriton Kalantidis

**Affiliations:** 1 Department of Biology, University of Crete, 70013 Heraklion, Greece; 2 Institute of Molecular Biology and Biotechnology, Foundation for Research and Technology-Hellas, 70013 Heraklion, Greece

**Keywords:** *Nicotiana benthamiana*, Transgene, Systemic post-transcriptional silencing, Photosynthesis, Defense response, Molecular biology, Plant sciences

## Abstract

Plants respond to biotic and abiotic stress by activating and interacting with multiple defense pathways, allowing for an efficient global defense response. RNA silencing is a conserved mechanism of regulation of gene expression directed by small RNAs important in acquired plant immunity and especially virus and transgene repression. Several RNA silencing pathways in plants are crucial to control developmental processes and provide protection against abiotic and biotic stresses as well as invasive nucleic acids such as viruses and transposable elements. Various notable studies have shed light on the genes, small RNAs, and mechanisms involved in plant RNA silencing. However, published research on the potential interactions between RNA silencing and other plant stress responses is limited. In the present study, we tested the hypothesis that spreading and maintenance of systemic post-transcriptional gene silencing (PTGS) of a GFP transgene are associated with transcriptional changes that pertain to non-RNA silencing-based stress responses. To this end, we analyzed the structure and function of the photosynthetic apparatus and conducted whole transcriptome analysis in a transgenic line of *Nicotiana benthamiana* that spontaneously initiates transgene silencing, at different stages of systemic GFP-PTGS. In vivo analysis of chlorophyll *a* fluorescence yield and expression levels of key photosynthetic genes indicates that photosynthetic activity remains unaffected by systemic GFP-PTGS. However, transcriptomic analysis reveals that spreading and maintenance of GFP-PTGS are associated with transcriptional reprogramming of genes that are involved in abiotic stress responses and pattern- or effector-triggered immunity-based stress responses. These findings suggest that systemic PTGS may affect non-RNA-silencing-based defense pathways in *N. benthamiana*, providing new insights into the complex interplay between different plant stress responses.

## Introduction

RNA silencing is a conserved mechanism of regulation of gene expression directed by small RNAs (sRNAs)^1–3^. Intracellular double-stranded RNAs (dsRNAs) are cleaved by RNase III-like endonucleases of the Dicer (DCR) family into sRNA, which are subsequently incorporated into an ARGONAUTE (AGO)-containing complex to guide the sequence-specific targeting of cognate RNAs. In some organisms, including plants, RNA-dependent RNA polymerases (RDRs) synthesize double-stranded RNAs from single-stranded RNAs to initiate silencing and amplify the silencing signal through the generation of secondary sRNAs. RNA silencing can take place at the transcriptional level (TGS) via methylation of DNA-cytosine bases by RNA-directed DNA methylation (RdDM) or at the post-transcriptional level (PTGS) either by cleavage or translational arrest of mRNA. In plants, RNA silencing plays a pivotal role in growth and development, response to abiotic stressors, repression of transposable elements, and defense against invading nucleic acids, including viruses and transgenes^2,4^.

Antiviral- and transgene-PTGS in plants are mechanistically and genetically related^1,2,4,5^. The *Arabidopsis thaliana* genome encodes 4 DICER-LIKE (DCLs), 10 ARGONAUTE, and 6 RNA-DEPENDENT RNA POLYMERASE proteins^1^. *Nicotiana benthamiana* presents a highly similar repertoire of key RNA silencing-encoding genes to Arabidopsis^6,7^. Virus-derived sRNAs are processed from dsRNA originating from replication intermediates and genomic secondary structure in RNA viruses and bi-directional transcription in DNA viruses. Transgene-derived siRNAs are processed from dsRNAs produced from transgenes expressed in sense (S), anti-sense (AS), and inverted-repeat (IR) orientations. dsRNAs from IR- and AS-transgenes are formed spontaneously upon transcription and occasionally upon annealing of the AS with the endogenous S-transcript, respectively. Aberrant uncapped products from read-through S-transgene transcription are substrates for RDR6 to synthesize dsRNA and initiate silencing^8^. Virus- and transgene-derived dsRNAs are targeted by the four DCLs, but the bulk is processed by DCL4 and DCL2 and bound to AGO1 and AGO2 for direct cleavage of transgene and virus RNAs. Potent viral- and transgene-PTGS both require the activity of DCL2, DCL4, and AGO1^6,8,9^. Additional proteins facilitating antiviral- and transgene-PTGS include the dsRNA-binding protein DRB4, which physically interacts with DCL4 to assist its slicing activity^10–14^. Once initiated in some cells, antiviral and transgene PTGS can spread to the entire plant through the vasculature and subsequently be maintained, leading to systemic viral resistance and transgene silencing. In *N. benthamiana*, both systemic viral resistance and transgene silencing require the activity of DCL2 and RDR6, as well as the production of secondary siRNAs^15,16^.

RNA silencing is the primary antiviral innate immune system in plants. The importance of RNA silencing in antiviral defense is supported by the prevalence of viral suppressors of RNA silencing (VSRs) in various virus genomes^17,18^. In addition to RNA silencing, other defense mechanisms play a pivotal role in the plant's resistance to virus infection. Aberrant viral RNAs can be recognized and degraded by the RNA quality control pathway^19,20^. Another defense mechanism pertains to the translational repression of host and viral RNAs^21–23^. Pattern- and effector-triggered immunity (PTI and ETI) are additional defense mechanisms playing pivotal roles in the plant's resistance to various pathogens, including viruses^21,22,24–27^. PTI downstream signaling involves activation of mitogen-activated protein kinases (MAPKs), calcium ion influx, production of reactive oxygen species (ROS), massive transcriptional reprogramming of genes involved in the production of antimicrobial compounds, defense hormones including salicylic acid (SA) and jasmonic acid (JA), and cell wall strengthening. In antiviral PTI/ETI, current knowledge supports that virally derived nucleic acids are recognized as PAMPs, and viral proteins, including coat proteins, movement proteins, and replicases, some also acting as VSRs, act as AVR factors. Chloroplasts play a critical role in the orchestration of plant defense responses due to their central role in energy production, ROS production, and the synthesis of defense hormones such as SA and JA^28–32^. Signaling between the chloroplast and nucleus coordinates the transcriptional up-regulation of defense genes and repression of photosynthetic genes and activity, leading to ROS production and potent hypersensitive response (HR)^33–35^. In addition, the decrease in photosynthetic activity frequently observed during virus infection is considered a defense mechanism to impede pathogen proliferation by reducing available carbon sources^28,30^.

Specifically, upon virus infection, the co-activation of multiple defense pathways and their interactions enable the mounting of a global and efficient response. Accumulating evidence supports the presence of synergistic and antagonistic interactions between antiviral PTGS and non-RNA silencing defense pathways^20,22,36–41^. Antiviral-PTGS and the RNA quality control pathways compete for the same substrates^19,20^. Constitutive repression of R gene expression controlled by AGO1 and RDR6 is released upon AGO1 inhibition by VSRs, leading to PTI/ETI-induced HR^42,43^. In addition, potent HR against the turnip crinkle virus requires DRB4, DCL4, and RDR6 functions^44^. ETI induction following virus recognition by a NBS-LRR entails AGO4-dependent translational repression of viral mRNAs^45^. Various hormones, including ABA and SA, and defense molecules such as ROS, modulate the expression level of RNA-silencing enzyme-coding genes^36^. In parallel, light intensity and photoperiod both control SAR and SA-mediated PTI/ETI via the phytochrome signaling pathway^46,47^. In turn, SA-induced expression of RDR1 has been shown to confer resistance to virus infection in *N. tabacum, N. benthamiana*, and *A. thaliana*^48–50^.

Currently, there is a lack of documentation on the potential consequences of systemic transgene silencing on cellular physiology and plant immunity. We have previously shown that light intensity is positively correlated with the induction of systemic PTGS in a transgenic line of *N. benthamiana* (GFP6.4) presenting spontaneous and stochastic systemic PTGS of a GFP transgene^51,52^. In the present study, we tested the hypothesis that, in the absence of stress conditions, the initiation and progression of systemic GFP-PTGS may induce changes in photosynthetic activity and gene expression patterns that pertain to non-RNA silencing-based antiviral responses. To this aim, we analyzed the structure and function of the photosynthetic apparatus and conducted whole transcriptome analysis in GFP6.4 plants at distinctive progressive stages of GFP-PTGS, namely before initiation, during ongoing spreading, and during maintenance of fully established systemic silencing. Photosynthetic activity in GFP6.4 plants before initiation and during maintenance of systemic GFP-PTGS was identical to that in wild type plants. However, whole transcriptome analysis revealed that a subset of genes involved in development, signal transduction, abiotic stress response and the PTI/ETI response were differentially expressed during the ongoing spreading and maintenance of systemic GFP-PTGS. Our results suggest that systemic transgene-PTGS crosstalk with non-RNA silencing-based defense pathways in *N. benthamiana*.

## Results

### Spontaneous and stochastic systemic GFP-PTGS in the *N. benthamiana* GFP6.4 transgenic line

**Figure 1:**
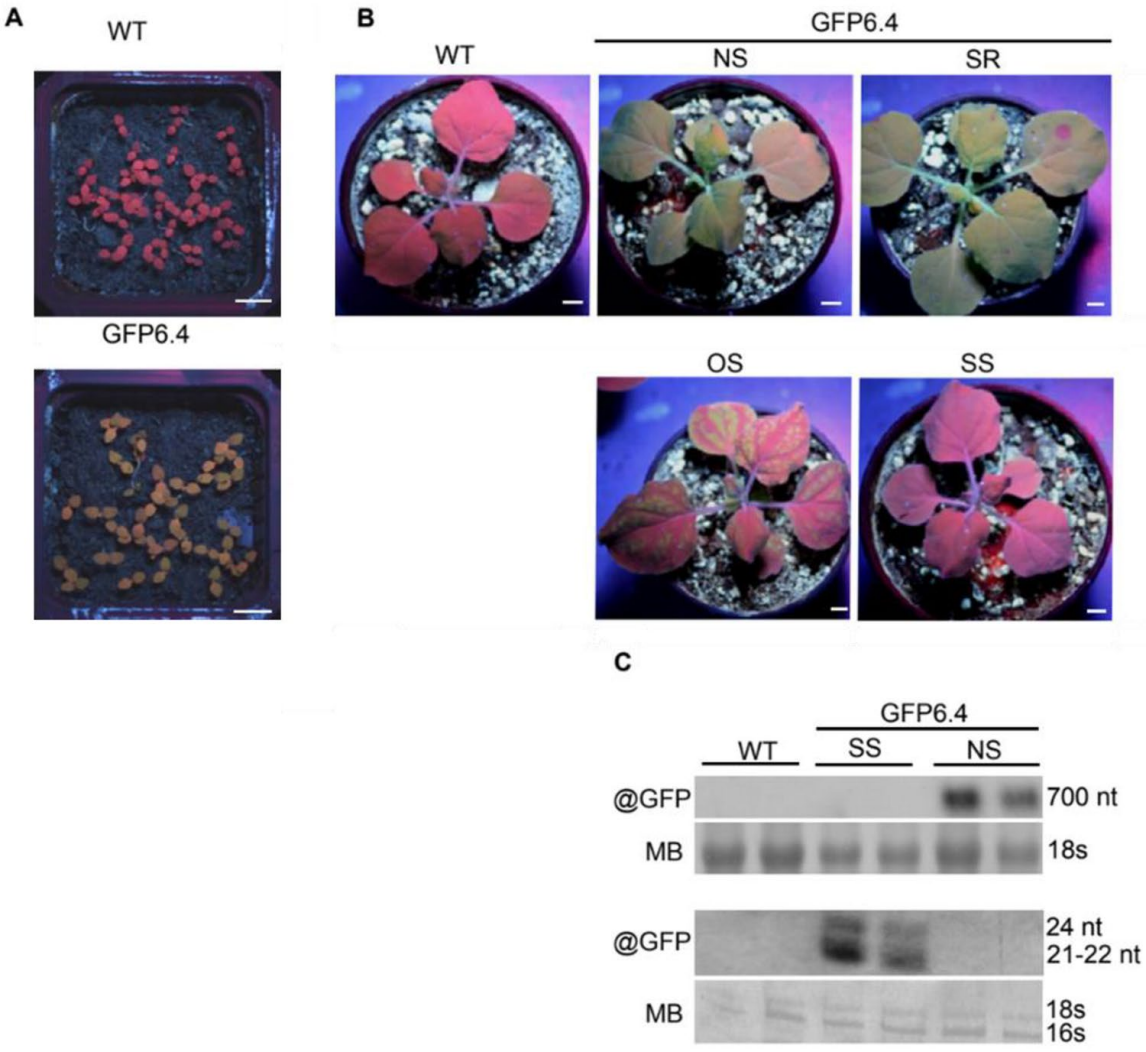
(**A**) GFP6.4 seedlings at the cotyledon stage, (**B**) Plants with no-silencing (NS), short-range (SR), ongoing spreading (OS), and maintained systemic silencing (SS) and (**C**) Northern blot analysis that shows the mGFP4-derived small RNAs of 21–24 nt in GFP6.4 SS.

*Nicotiana* *benthamiana* transgenic line GFP6.4 was generated by *Agrobacterium tumefaciens*-mediated transformation of a T-DNA carrying the *nptII* gene under the control of the nopaline synthase promoter and conferring resistance to kanamycin and the *mGfp4* gene under the control of the cauliflower mosaic virus (CaMV) *35S* promoter^51,52^. GFP6.4 homozygous lines were created by propagating GFP6.4 plants from seed. GFP fluorescence was readily visible under UV light in every GFP6.4 seedling at the cotyledon stage (Fig. 1A). From the first two leaves, local short-range GFP silencing was visible as red foci of chlorophyll fluorescence that eventually appeared in a spontaneous and stochastic manner in a subset of plants. GFP silencing subsequently spread along the vasculature to the entire plant and was maintained for the entire plant's life span. As the experiment progressed, GFP silencing eventually initiated in every plant, albeit at a different time, and all plants were fully silenced before reaching senescence. The progeny of GFP-silenced plants was all GFP-fluorescent at the cotyledon stage, indicating that silencing was abolished during gamete formation or embryogenesis. While the initiation of GFP silencing was stochastic, its occurrence, progression, and transmission were nevertheless deterministic, i.e., it occurred in every plant, was unidirectional from systemic spreading to maintenance, and was reset at each generation. Under our experimental growth conditions, GFP6.4 plants having reached the stage of four to six leaves included plants presenting no-silencing (NS), short-range (SR), ongoing spreading (OS), and maintained systemic silencing (SS) with comparable number of individual plants in each phenotypic class (Fig. 1B). GFP6.4 NS, OS, and SS plants were indistinguishable in terms of size and continued to develop at a comparable pace. Northern blot analysis using a probe homologous to the mGFP4 sequence revealed the presence of mGFP4-derived small RNA of 21–24 nt length in GFP6.4 SS plants, indicating that GFP silencing was achieved by post-transcriptional cleavage of the mGfp4 mRNA (Fig. 1C). Progeny of GFP6.4 plants out-crossed to WT plants (i.e., F2 progeny of WTxGFP6.4 F1 plants) segregated at 90%-93% for both kanamycin resistance and GFP fluorescence, suggesting the presence of two unlinked T-DNAs expressing both a functional *nptII* and mGFP4 transgene in the GFP6.4 line (Supplementary Table 1). Southern blot hybridization with a probe homologous to the mGFP4 sequence confirmed the presence of two copies of the mGFP4 transgene in the GFP6.4 line^51^. GFP6.4xWT F1 seedlings were all kanamycin-resistant and GFP-fluorescent, albeit at a reduced level compared to the GFP6.4 parental line. None of the GFP6.4xWT F1 plants, however, developed GFP-PTGS, suggesting that homozygosity of at least one of the two mGFP4 alleles was required for the onset of GFP-PTGS. The presence of two unlinked and functional mGFP4 alleles and the requirement for the homozygosity of at least one allele for GFP-PTGS to occur suggest that GFP-PTGS in the GFP6.4 line is achieved by S-PTGS. These plants present a genetically homogenous but, as far as S-PTGS is concerned, heterogeneous population to identify hidden differences between the NS-SS and OS phenotypic subgroups.

### Photosynthetic activity in systemic GFP-PTGS

**Figure 2:**
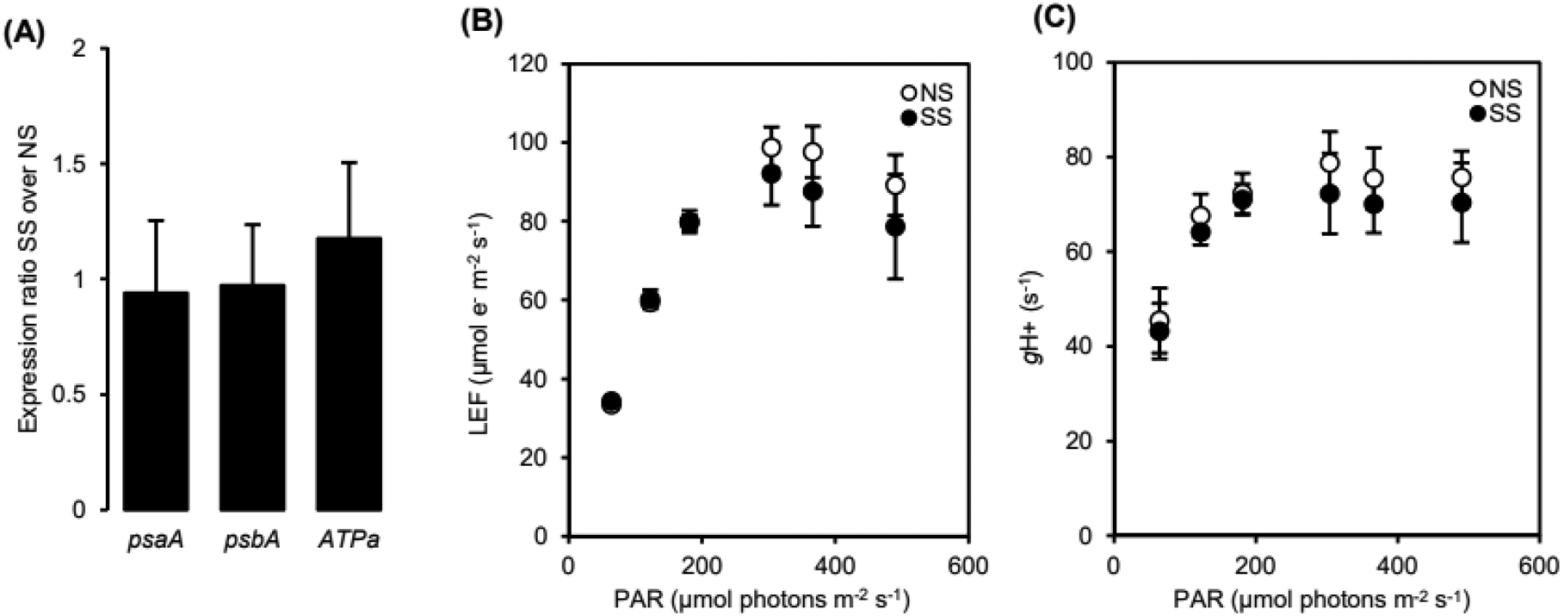
(**A**) qPCR-relative expression levels of chloroplast-encoded genes involved in the photosynthetic electron transport chain, (**B**) photosynthetic linear electron flow (LEF)and (**C**) the apparent proton conductivity of the CF1–CF0 ATP synthase (gH+).

Plant responses to virus infection are frequently associated with a reduction in photosynthetic gene expression and activity. To characterize the possible consequences of GFP-PTGS on the photosynthetic activity of the GFP6.4 line, we first compared the structure and function of the photosynthetic apparatus in GFP6.4 NS and SS plants at steady-state. The relative expression levels of chloroplast-encoded genes involved in the photosynthetic electron transport chain were determined using quantitative RT-PCR (qRT-PCR) (Fig. 2A). Relative transcript abundances of genes coding for PSI P_700_ chlorophyll and apoprotein A1 (psaA), PSII protein D1 (psbA), and the A subunit of the catalytic component (CF1) of the proton-linked Adenosine triphosphate synthase (ATPa) were determined. Further insights into the photosynthetic activity were gained by analyzing the photosynthetic linear electron flow (LEF) and the proton conductivity of the CF1-CF0 ATP synthase (gH+) by conducting in vivo measurements of chlorophyll fluorescence yield and electrochromic shift decay, respectively (Fig. 2B,C). GFP6.4 NS and SS plants presented similar values for both LEF and gH+. The OJIP test analysis of chlorophyll fluorescence transients is a quick approach that yields a set of parameters from which a Performance Index on Absorption Basis (PI_ABS_) is calculated, providing information on the structure and function of electron transport chains^53,54^. The OJIP-test is commonly used to study the effects of virus infection and abiotic stresses on plant photosynthetic activity^55,56^. To detect possible transitory adjustments of the photosynthetic structure and function that may take place at the onset and progression of GFP-PTGS, we conducted a time series analysis of the OJIP-test parameters in WT and GFP6.4 plants from the NS to the SS stages (Supplementary Fig. 1). GFP6.4 and WT plants presented both a similar trend in PIABS over time and comparable values in PIABS during the entire course of the experiment, irrespectively of the onset, spreading, and maintenance of GFP-PTGS. Taken together, these results indicate that systemic GFP-PTGS does not significantly affect the apparent structure and function of the photosynthetic apparatus in the GFP6.4 line.

### Alterations of biotic and abiotic stress related genes’ expression in transgenic GFP6.4 plants

Plant response to virus infection involves an intense transcriptional reprogramming of protein-encoding genes of various functions, including defense and stress responses. To characterize the specificities of the transcriptional reprogramming taking place in GFP6.4 NS, OS and SS versus WT plants, up- and down-regulated gene sets were separately subjected to Venn diagram analysis (Supplementary Fig. 2). The number of differentially expressed genes was calculated by combining the DESeq2 and edgeR analyses. Each sample had between 9 and 11 million reads sequenced (Supplementary Table 2). They were then mapped onto the *N. benthamiana* v1.0.1 Sol Genomics database (https://solgenomics.net/organism/Nicotianabenthamiana/genome). SS plants presented the largest proportion of significantly DEGs (Log2 fold change ≥ 1.5 and p-value cutoff = 0.05) i.e., 12.9% in NS (54 out of 419), 12.1% in OS (11 out of 91) and 25% in SS (65 out of 260) implying that SS is associated with more pronounced transcriptional reprogramming than NS and OS groups.

**Figure 3:**
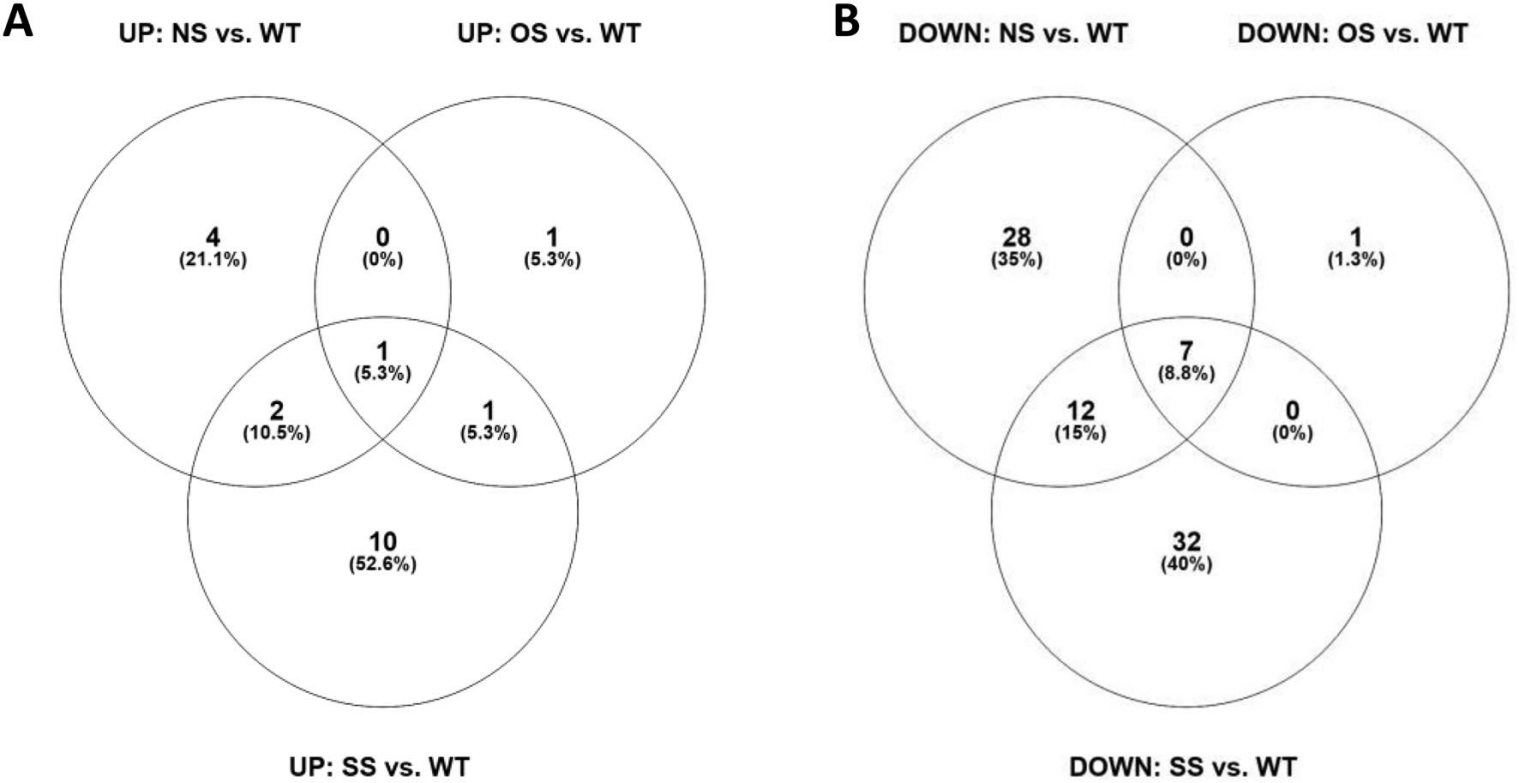
Number of genes that are significantly differentially expressed (Log2 fold change ≥ 1.5) in the transcriptome of GFP6.4 NS, OS, and SS groups when compared to WT plants. (**A**) Upregulated genes in GFP6.4 plants compared to WT and (**B**) Downregulated genes in GFP6.4 plants compared to WT.

Some of the expressed genes were also found significantly differentially expressed (DEGs) across all GFP6.4 groups versus WT plants, indicating that the GFP6.4 transgenic plants could be differentiated from the WT plants at a transcriptional level, regardless of the PTGS state. To identify these genes, all GFP6.4 NS, OS and SS DEGs were subjected to Venn diagram analysis, which revealed the shared genes that are significantly differentially expressed in all groups versus WT (Fig. 3 and Table 1). Based on the gene IDs, we determined the name and function of each gene. Table 1 lists the genes that are differentially expressed in all three states of GFP transgenic plants when compared to WT plants.

**Table 1:** Differentially expressed genes that are common in all three states of the GFP transgenic plants, compared to wild-type plants.

NS, OS, SS versus WT			
ID/Name		Gene expression	Log2 fold change
Niben101Scf01578g00012.1	GASA/GAST/Snakin	Upregulated	NS: 1.979 OS: 1.718 SS: 1.854
Niben101Scf03870g01016.1	Homeobox-leucine zipper protein family (HD-Zip)	Downregulated	NS: − 10.144 OS: − 10.253 SS: − 8.53
Niben101Scf09044g01012.1	Thaumatin-like protein	Downregulated	NS: − 2.742 OS: − 1.713 SS: − 2.313
Niben101Scf00472g06013.1	Ribosome biogenesis protein BOP1 homolog	Downregulated	NS: − 2.222 OS: − 1.849 SS: − 1.925
Niben101Scf02982g01023.1	Chaperone protein DnaJ	Downregulated	NS: − 2.119 OS: − 1.786 SS: − 2.612
Niben101Scf08508g02011.1	F-box protein PP2-A13	Downregulated	NS: − 1.652 OS: − 1.486 SS: − 2.597
Niben101Scf08799g00001.1	RPM1 interacting protein 4 (RIN4) transcript 2	Downregulated	NS: − 1.59 OS: − 1.862 SS: − 1.633
Niben101Scf01959g01001.1	Unknown product [*Oryza sativa* Japonica Group]	Downregulated	NS: − 2.464 OS: − 1.731 SS: − 2.793

Our transcriptomic analysis revealed that only one gene, which belongs to the Gibberellic Acid Stimulated Arabidopsis (GASA) gene family, was significantly upregulated in all three PTGS states versus WT. In regard to the downregulated genes, we found seven genes that were significantly downregulated in all three PTGS states of the GFP6.4 plants, compared to WT. Six of them have been previously characterized. Independently of the silencing state, all six genes are involved in stress response pathways. More specifically, we identified the downregulation of: (1) the transcription factor ATHB13 from the homeodomain-leucine zipper (HD-Zip) protein family, (2) a protein coding gene from the Thaumatin-like family (TLP), (3) Resistance to *Pseudomonas syringae pv. maculicola* 1 (RPM1)-Interacting Protein 4 (RIN4) protein, a member of the intracellular nucleotide-binding leucine-rich repeat receptor (NLR) class of R proteins, (4) a gene that is required for the maturation of ribosomal RNAs, called Block of Proliferation 1 (BOP1), (5) a DnaJ protein, which belongs to the DnaJ protein family, the members of which act as key regulators of protein homeostasis, and (6) the F-box/lectin family protein, known as Phloem Protein2-A13 (PP2-A13).

### Both spreading and maintenance of systemic PTGS affect plant stress related genes in GFP transgenic plants

Next, we focused on the transcriptional differences between the GFP transgenic plants in different silencing states, in order to identify differentially regulated genes among genetically identical plants (Supplementary File 1). To test the hypothesis that the transgene's PTGS spreading and maintenance are associated with transcriptional changes related to non-RNA silencing-based stress responses, we used GFP6.4 plants that express the GFP transcript but are in different PTGS states (NS, OS and SS). For this, we compared the GFP6.4 OS and SS transcriptomes separately to the GFP6.4 NS transcriptome. It should be noted that these plants are clones, with the only difference being the stochastic timing of transgene silencing expression. Therefore, we considered conducting a broader analysis that would include all candidates with a Log2FoldChange of 1 and a p-value cutoff of 0.1, in order to detect even minor but significant transcriptional differences. Table 2 shows all the significantly differentially expressed genes between the GFP6.4 NS and the OS and SS transcriptomes.

**Table 2:** Differentially expressed genes between GFP6.4 NS to the OS and SS transcriptomes.

ID/name		Gene expression	Log2 fold change	qPCR verification
OS versus NS				
Niben101Scf01374g07013.1	CASP-like protein (DUF1677)	Upregulated	6.051	–
Niben101Scf07121g03008.1	Peroxidase 15	Downregulated	− 20.64	✓
Niben101Scf07937g01006.1	BnaA05g03240D [*Brassica napus*]	Downregulated	− 4.213	–
SS versus NS				
Niben101Scf04574g08003.1	Tetratricopeptide repeat (TPR)-like superfamily protein	Upregulated	4.581	✓
Niben101Scf07516g04001.1	Cytochrome P450 superfamily protein	Upregulated	4.237	✓
Niben101Scf06080g03009.1	C2 calcium/lipid-binding plant phosphoribosyltransferase family protein	Upregulated	3.218	✓
Niben101Scf15051g00012.1	Protein kinase superfamily protein	Upregulated	2.911	✓
Niben101Ctg13703g00001.1	UDP-Glycosyltransferase superfamily protein	Upregulated	1.29	N/A
Niben101Scf08704g01003.1	Stress responsive A/B Barrel Domain	Downregulated	− 1.062	–
Niben101Scf03768g00007.1	Protein RESPONSE TO LOW SULFUR 1 (*A. thaliana*)	Downregulated	− 2.944	✓
Niben101Scf06026g01003.1	WRKY transcription factor 44	Downregulated	− 2.612	N/A

Although our transcriptomic analysis included three technical and four biological replicates, we opted to further verify the transcriptional differences observed by RT qPCR. Since the NS, OS, and SS groups consist of genetically identical plants, we were interested in confirming the levels of DEGs in the OS and SS groups versus the NS group. Hence, we focused on the genes that showed altered expression within the different silencing states for the qPCR verification. The graphs presented in Supplementary Fig. 3 and the data in Table 2 indicate the genes for which differential expression was also verified by qPCR, confirming our RNA-Seq results. In Supplementary Fig. 3, the results are presented as relative expression levels. This analysis showed that in all but three cases RT qPCR confirmed the RNAseq results.

### Differentially expressed genes: NS and OS–PTGS groups

Three genes are significantly differentially expressed in the OS group of transgenic plants when compared to the NS group: (1) a putative CASPARIAN STRIP MEMBRANE DOMAIN PROTEIN (CASP-like protein, DUF1677), (2) a PEROXIDASE15 gene, and (3) the unidentified BnaA05g03240D gene. Specifically, the CASP-like protein, likely involved in the Casparian strip formation, is upregulated. On the other hand, our data indicate a strong downregulation of the PEROXIDASE 15 gene (PER15), member of the plant peroxidase family and also related to the Casparian strip formation^57^.

### Differentially expressed genes: NS and SS-PTGS groups

A total of eight different genes were identified as differentially expressed between NS and SS GFP6.4 plants, and all of them have been linked to stress responses. Significantly upregulated are: (1) a Tetratricopeptide Repeat (TPR)-like protein, (2) a Cytochrome P450 gene, (3) a C2 calcium/lipid-binding plant phosphoribosyltransferase, and (4) a protein kinase. Further, significantly downregulated are: (1) the AT5g22580.1, identified in *A. thaliana*, that corresponds to a stress responsive a/b barrel domain, (2) the Response to Low Sulfur Protein 1 (LSU1) and (3) the transcription factor WRKY44.

## Discussion

There are numerous mechanisms and pathways that have pivotal role in plant immunity. Co-activation of multiple defense pathways and their interactions during biotic or abiotic stress allow for an integrated and efficient response. Antiviral and transgene systemic PTGS share genetic and mechanistic similarities. In the current study, we used GFP6.4 transgenic *N. benthamiana* plants, a line that expresses GFP and then spontaneously and stochastically silences transgene expression. We took advantage of this system to ask whether, in this specific genetic background, the non-phenotypically initiated, ongoing, and systemically active state of silencing affects gene expression, and eventually leads to altering cellular physiology. We first showed that GFP-PTGS has no impact on the structure and function of the photosynthetic apparatus in the GFP6.4 transgenic plants. Next, we found that GFP transgene expression may be associated with altered levels of defense and development-related genes, at the transcriptional level, regardless of the GFP silencing state. We also recorded that, in the absence of obvious abiotic stress and pathogen infection, the OS and SS states of GFP-PTGS were both associated with transcriptional changes in genes primarily involved in defense and stress response pathways.

### Systemic GFP-PTGS incurs changes in whole transcriptome profiles

Sohn et al.^58^ published a whole transcriptome analysis in silenced and non-silenced leaf regions of a *N. benthamiana* transgenic line exhibiting local cell-autonomous leaf-partitioned GFP silencing achieved through both PTGS and TGS mechanisms. In this study, leaf region-specific DEGs were three times more abundant in non-silenced than in silenced regions. In addition, the DEG set specific to non-silenced regions included various genes involved in nucleic acid metabolism, stress response, and defense, suggesting that transgene expression may lead to changes in transcriptional activity of specific genes.

The same authors found that genes involved in RNA silencing pathways were expressed similarly in non-silenced and silenced leaf regions. Likewise, in our study no differences in the expression levels of genes involved in RNA silencing pathways were detected between the NS, OS, and SS GFP6.4-PTGS states and the WT state in our transcriptome analysis. As a result, cell-autonomous and systemic transgene silencing may not be restricted by the relative abundance of RNA silencing enzymes. However, our analysis of GFP6.4 the largest proportion of DEGs in the SS transcriptome than in the OS and NS transcriptomes. Consequently, systemic GFP-PTGS appears to have a greater transcriptional effect than stable GFP expression in the GFP6.4 transgenic line. This discrepancy may be due to differences in GFP transgene insertion loci or copy number between our GFP6.4 transgenic line and the leaf-partitioned GFP silencing transgenic lines. Alternatively, the systemic nature of GFP-PTGS in GFP6.4, rather than local silencing in the partitioned transgenic line, could be responsible for the transcriptional reprogramming observed in the SS plants.

### GFP transgene expression alone may be associated with mildly disrupted basic cell processes and stress responses

In our study, we looked into whether the GFP6.4 transgenic plants could be differentiated from the WT plants at a transcriptional level, regardless of their silencing state. To this end, we compared the transcriptomes of GFP6.4 NS, OS, and SS groups to that of WT plants and identified the shared DEGs among all silencing states versus WT. Only one gene, from the Gibberellic Acid Stimulated Arabidopsis (GASA) gene family, was found upregulated in the transgenic plants. Generally, the GASA family is of great importance for plant growth, development, and stress response. It's interesting to note that numerous studies in various plants species demonstrate the role of the GASA genes in plants' responses to both biotic and abiotic stress^59–61^. According to the literature, overexpression of GASA4 from beechnut (*Fagus sylvatica*) enhances tolerance to salt, ROS, and heat stress in transgenic Arabidopsis plants^59^. In addition, *Rhizoctonia solani* and *Erwinia caratovora* resistance in transgenic potato plants was found to be increased by overexpression of the GASA-like gene Snakin-1^60^. Snakin-2 inhibits *Clavibacter michiganensis* in tomatoes via controlling the redox levels^61^. Given that GASA genes are involved in plant responses to both biotic and abiotic stress in several plant species, its overexpression in all silencing states of GFP6.4 plants suggests that they may perceive transgene expression as a stressor even in the absence of obvious stress conditions.

On the other hand, in all three groups of the GFP transgenic plants, pathogenesis-related genes appear downregulated compared to the WT. To get beyond innate immunity, many plant pathogens employ effector proteins that are delivered into host cells. However, plants’ disease resistance (R) proteins or pathogenesis-related proteins (PRs) serve as effector recognition mechanisms^62^. Specifically, two pathogenesis-related proteins are suppressed in the GFP6.4 plants: a Thaumatin-like protein (TLP) and RPM1-Interacting Protein 4 (RIN4). TLPs have been classified as the fifth family of pathogenesis-related proteins (PR-5), according to Van Loon et al.^63^. TLPs act against oomycetes, while RIN4 is an important plant immunity regulator, present and well-studied in many plant species. RIN4 responds to effectors by inducing a typically strong immune response known as NLR-triggered immunity (NTI)^64^. Particularly, the downregulated RIN4 likely acts as an immune-signaling hub^65^. This de-regulation of pathogenesis-related genes in GFP6.4 plants could indicate that transgene expression itself may be perceived as a stress factor by the plant.

A number of genes involved in cell homeostasis are also suppressed in all GFP6.4 plants compared to the WT. For instance, the ATHB13 gene, which belongs to the HD-Zip family of transcription factors, and is unique to plants, appears to be significantly downregulated^66^. The *A. thalian*a genome contains 47 HDZip genes, grouped into four different classes: HDZip I to IV. Among them, HDZip class I proteins are widely expressed in all plant organs, and they activate gene expression^67^. ATHB13 gene is a member of the HDZip I class, the members of which are recognized to help with abiotic stress responses and tolerance^68^. As far as we know, ATHB13, potentially acts as a mediator of sucrose signaling^69^ and its suppression can cause developmental defects^70^.It is worth noting that, while members of the HDZip I class have been shown to help in abiotic stress responses and tolerance, it's not uncommon for genes from the same family to compete for the same substrates. Suppressing a less efficient member of a gene family can frequently result in improved efficiency of the other members. This has previously been noticed and reported in studies such as in Katsarou et al.^9^. While there is no solid evidence in the present case, it would not be surprising to discover that members of the same gene family play opposite functions. Furthermore, BLOCK OF PROLIFERATION 1 (BOP1), which facilitates ribosome maturation, is conserved among eukaryotes and it is also suppressed in GFP6.4 NS, OS, and SS groups compared to the WT plants. *A. thaliana* BOP1 downregulation strongly impacts cell division^71^ and the suppression of its orthologue FvBOP1 in strawberry plants results in increased anthocyanin levels, premature senescence and incapability of fruit production, indicating the induction of stress response^71,72^. Moreover, our data show the downregulation of a DnaJ protein. DnaJ proteins, often referred to as heat-shock protein 40 (HSP40), are related to heat stress responses. They act as molecular chaperones either on their own or in conjunction with HSP70^73^. This is another clue that suggests abnormalities in basic cell processes, as J-proteins are implicated in heat stress responses in several plant species^74–76^. Finally, the F-box/lectin family protein, known as PHLOEM PROTEIN2-A13 (PP2-A13), is also reduced among all three PTGS states of the GFP6.4 transgenic plants. Recent research by Liu et al.^77^ showed that the expression of PP2-A13 is photoperiodically triggered and necessary for plant fitness in winter, when the length of day time is shorter than the length of night time.

Our findings suggest that expression of the GFP transgene may mildly disrupt basic cell processes as well as plant defense and stress responses. However, since position effects originating from transgene insertion could interfere with the interpretation of our results, we further focused on comparing the different states of silencing in the same genetic background (GFP6.4 transgenic plants). Nevertheless, the fact that the vast majority of genes affected by the transgenic event fall into the stress response category could be a plausible hint that these changes may be rooted in the transgenesis or transgene itself rather than the position of the insertion.

### Both spreading and maintenance of systemic GFP-PTGS are associated with transcriptional alteration of genes involved in plant defense and stress responses

In our study, we have also compared GFP6.4 plants in different PTGS stages to test the hypothesis that spreading and maintenance of PTGS of the GFP transgene are associated with transcriptional changes that pertain to non-RNA silencing-based stress responses. For this, we separately compared the transcriptomes of the OS and SS plant groups to that of the NS plants. For this transcriptomic analysis we chose a p-value cutoff of 0.1 for the DEG comparison of genetically identical plants, carefully considering our research question and study design, as well as the potential consequences of making Type I and Type II errors. A p-value cutoff of 0.1 is a rather permissive p-value threshold in contrast to the standard cutoff of 0.05, usually employed in RNA-seq experiments. However, in the case of identifying the DEGs among genetically identical plants, using a p-value of 0.1 instead of 0.05 while considering as significantly differentially expressed genes the ones with a log2 fold change greater than or equal to 1.5 can be very informative. Our study addresses a research question that has not been previously investigated in depth, and there is no specific prior knowledge to support it. To this end, we introduce a broader concept that can serve as a framework for future research into new subjects as well as enable other researchers to pursue the issue further in the future. Additionally, given that our sample size could be regarded as small, a less stringent p-value threshold may help increase the chances of detecting potentially interesting effects. We have considered that the consequences of failing to detect a true effect (a Type II error) are more severe than the consequences of falsely detecting an effect (a Type I error) in the statistical analyses.

A comparison of the transcriptomes of the OS and NS groups revealed three differentially expressed genes. According to the literature, only two of these have been previously characterized (a putative CASPARIAN STRIP MEMBRANE DOMAIN PROTEIN and PEROXIDASE 15). First and foremost, comparing OS to NS plants revealed an increase in the expression of a putative CASPARIAN STRIP MEMBRANE DOMAIN PROTEIN (CASP-like protein, DUF1677). CASPs are a large family of proteins that are expressed in a tissue-specific manner. Robert Caspary discovered the Casparian strip, a ring-like cell wall structure found in the root endodermis of vascular plants. The root endodermis is responsible for nutrient uptake and stress resistance^78^. In order to facilitate the deposition of Casparian strips in the endodermis, CASPs enlist the lignin polymerization apparatus^78,79^. Consequently, changes in the expression of CASP genes may lead to alterations in root formation in response to several abiotic stresses^80–82^.

On the other hand, our data indicated a strong downregulation of the PEROXIDASE 15 gene (PER15), a member of the plant peroxidase family, in the OS group. *A. thaliana's* genome encodes 73 peroxidase genes with complex regulation patterns that are affected by a variety of biotic and abiotic factors at different times and locations^83^. Peroxidases are also implicated in lignin deposition upon the Casparian strip formation^57^. It is currently known that plants can correctly form transcellular barrier networks by the spatially exact deposition of specific cell wall components using members of the Casparian Strip Integrity Factors (CIF) family. According to Rojas-Murcia and colleagues (2020) PER15 and PER49 were two of the most highly upregulated genes in response to CIF1 and CIF2 stimulation. Increased lignification in the cell corners of Casparian strip mutants appears to be directly related to increased ROS generation and activated peroxidase gene expression^57^. We can speculate that down-regulation of PER15 may reduce the lignin content in the root cell walls, which may affect the plant’s resistance to biotic and abiotic stresses.

Therefore, when we compare the OS to the NS group of GFP6.4 transgenic plants, we identify altered expression of genes that are primarily implicated in root formation and could possibly affect plant stress responses and tolerance in the long run via regulation of root lignin deposition. The fact that the OS state has so few differences from the NS state is also intriguing, implying that the ongoing spread of silencing may be perceived by the plant as a similar state to no transgene silencing.

Transcriptome comparisons of SS and NS plants revealed that all of the highly differentially expressed genes between these silencing states are also involved in plant defense and stress responses. It is worth noting that in this case, we discovered a higher number of genes with altered expression than when comparing the OS with the NS group of GFP6.4 transgenic plants. In comparison to the NS group, SS plants have higher expression of five genes involved in signaling pathways and lower expression of three abiotic stress-related genes.

All genes found upregulated in the SS plants versus the NS plants are somehow involved in signal transduction. A TPR-like protein, containing a tetratricopeptide repeat (TPR) motif^84^, appears to be upregulated in SS transgenic plants when compared to the NS group. TPR proteins are involved in osmotic stress response and plant hormone signaling control, including ethylene production and gibberellin and cytokinin signaling during plant development^85–87^. Moreover, a Cytochrome P450 protein gene is upregulated in SS plants. The Cytochrome P450 (CYP) superfamily is the biggest enzymatic protein superfamily in plants, found in all kingdoms. Plant CYPs can absorb light at 450 nm and affect growth and development since they are involved in the biosynthesis of crucial secondary metabolites^88^. Through their implication on secondary metabolism, they protect plants under biotic and abiotic stress conditions^89^. Further, a C2 calcium/lipid-binding plant phosphoribosyltransferase family protein is another stress-related protein that is upregulated in the SS plants versus the NS. Studies have shown that calcium-binding proteins play a role both in biotic and abiotic stress signal transduction as well^90,91^. Next, a protein kinase family protein is also upregulated in the SS plants versus the NS. This group of kinases is part of a larger group of homologous kinases that participate in various physiological signaling cascades due to the crucial role they play in protein phosphorylation. For instance, research on Arabidopsis protein kinases has demonstrated how they control growth signaling pathways^92^ and their role in plant reproduction processes^93^. Additionally, protein kinases play a crucial role in how plants react to abiotic stress^94^. Besides that, they regulate signaling pathways that are activated in response to viral infections. Geminiviruses appear to alter the host's genes' expression through interactions with protein kinases in signal transduction pathways^95,96^.

Plants also have a class of stress-related proteins that contain the stress-response a/b barrel domain, which assembles into a dimer that is very stable^97^. Arabidopsis At5g22580.1 that corresponds to a stress responsive a/b barrel domain is downregulated in the SS transgenic plants when compared to the NS plants. Its specific function remains elusive. In *Populus balsamifera*, salt stress causes an upregulation of the stress-response a/b barrel domain^98^. Stress-responsive a/b barrel domain containing protein genes are also upregulated in response to drought conditions in *Brassica juncea*^99^. Furthermore, Response to Low Sulfur Protein 1 (LSU1) is downregulated in the SS group of transgenic plants. We currently only know that sulfur deficiency activates the *A. thaliana* LSU1, LSU2, and LSU3 genes, but their precise roles are unknown^100^. Large-scale plant interactome studies have revealed that the LSU protein family functions as a stress-related hub, integrating abiotic and biotic stress responses^101^. Lastly, our data show downregulation of a WRKY transcription factor, namely WRKY44. WRKY transcription factors are plant-specific transcriptional regulators. WRKY44 is produced by the TRANSPARENT TESTA GLABRA2 (TTG2) gene and it is the last downregulated gene in our dataset. This gene controls trichome development, mucilage and tannin production in seed coats, and possibly root hair development^102^. It has also been reported that WRKY44 inhibition regulates sugar metabolism and signaling and thus plays a role in drought response^103^. In Actinidia sp., WRKY44 is thought to be an anthocyanin pathway activator^104^.

The higher number of differentially expressed genes between SS and NS, compared to the very few differences between OS and NS, suggests that fully established systemic GFP silencing is associated with a larger transcriptional response than ongoing PTGS or stable GFP expression. In PTGS, it’s probable that GFP transcription remains unaffected. However, the final protein produced is impacted. Defining stressors in this context seems challenging, as one stressor might be the level of GFP protein or mRNA, while another could be the active targeting of a transcript for degradation. Such an event could be perceived as an ongoing threat. Given that all of the altered genes are linked to plant stress responses, we are tempted to speculate that transgene silencing may act as a minor yet perceivable stress factor for the transgenic plant.

## Materials and methods

### Plant material and culture

Wild type and transgenic GFP6.4 *N. benthamiana* seeds were sown in potting soil. The plantlets were grown in growth chambers under a 16-h/8-h light/dark photoperiod at 22 °C and a photosynthetically active radiation of 90 µmol m^−2^ s^−1^ provided by cool white fluorescent tubes. We used a hand-held 1000 W long-wavelength UV lamp (B1000 AP; Ultraviolet Products, Upland, CA, USA) to monitor the GFP fluorescence. We collected and analyzed four plant samples for each of the following groups: Wild Type (WT) plants and transgenic Non-Silenced (NS), Ongoing Spreading (OS) PTGS, and maintained Systemically Spread (SS) PTGS.

### Equipment and settings

Northern blot images (Fig. 1C) were acquired with a regular scanner using default parameters. The images were processed in PowerPoint by: "Crop" to select the samples WT, NS and SS, "Inverse Grayscale" and also slight changes were made in "Brightness" and "Contrast" settings and applied to the entire image. Supplementary information is included in the “Blot Images_Supplementary Information.pdf” file.

### In vivo measurement of photosynthetic activity

Apparent conductivity of the thylakoidal ATPase to protons (gH +  = 1/τ) was estimated by electrochromic shift measurements as in Ref.^105^. Absorbance changes at 520 nm were measured with an in vivo spectrophotometer as in Ref.^106^. Fitting curves for the estimation of tau (τ) were performed in Origin. The instrument was also used to measure changes in chlorophyll a fluorescence yield by using the 520-nm light-emitting diode bank as a probe beam, as described previously in^107^. Saturation pulses (> 7 000 µmol of photons m^−2^ s^−1^) were imposed by using light from the two red actinic LEDs, filtered through heat-absorbing glass. Actinic light was filtered out by using an RG-695 Schott glass filter. Saturation pulse-induced fluorescence yield changes were interpreted as described in Ref.^108^. More particularly, linear electron flow (LEF) was calculated by the following formula LEF = 0.84 × PAR ×  (Fm′ − Ft)/Fm′) where Fm′ is the maximal fluorescence value of a light adapted leaf after a saturating pulse, Ft is the level of fluorescence immediately before the saturating pulse and PAR is the photosynthetically active radiation in µmol of photons m^−2^ s^−1^.

### RNA isolation

For RNA isolation, 500 mg of homogenized plant tissue were used per *N. benthamiana* leaf sample. RNA extraction buffer (38% saturated phenol, 0.8 M guanidine thiocyanate, 0.4 M ammonium thiocyanate, 0.1 M sodium acetate, 5% glycerol) was added to each sample. Total RNA was extracted as described in Ref.^9^. RNA quality was evaluated after running a denaturing agarose gel (1% agarose, 0.7% formaldehyde, 1 × MOPS, 7 μg/100 ml ethidium bromide), in denaturing buffer (0.7% formaldehyde, 1 × MOPS).

### Northern blot hybridization

Northern blot analyses were conducted as described in Ref.^51^. DNA probe corresponding to the full length mgfp4 sequence was radio labeled by random priming using alpha-dCTP32 (Hartmann Analytic, Steinriedendamm, Braunschweig, Germany), random hexamers (Invitrogen™, ThermoFisher Scientific, Waltham, MA, USA) and Klenow fragment (Minotech Biotechnology, Heraklion, Crete, Greece). Detection of mgfp4 mRNAs and siRNAs were conducted on 10 µg and 50 µg of total RNAs respectively. Membranes were stained with methylene blue to control RNA loading. Hybridizations were conducted at 65 °C and 42 °C for the detection of mgfp4 mRNAs and siRNAs respectively.

### RNA sequencing and bioinformatic analysis

A total of 500 ng RNA per biological replicate was used for 3′ RNA sequencing, which was performed at the Genomics Facility of the Institute of Molecular Biology and Biotechnology of the Foundation for Research and Technology Hellas (IMBB-FORTH). The QuantSeq (https://www.lexogen.com/) was used. Following first-strand synthesis and RNA removal, the complementary strand was randomly primed (second-strand synthesis). The primers insert Illumina platform-specific linker sequences. The resulting double-stranded cDNA was purified using magnetic beads. The complete sequences required for cluster generation are then introduced by library PCR amplification. To analyze the transcriptional alterations that take place in GFP-PTGS, we used the R software (R Core Team, 2017), Bioconductor^109^ packages including DESeq2 and edgeR^110–112^ and the SARTools package for differential analysis of RNA-Seq count data, developed at PF2—Institut Pasteur. Normalization and differential analysis were carried out according to both DESeq2 and edgeR model and package. The statistical analysis process included data normalization, graphical exploration of raw and normalized data, test for differential expression for each feature between the conditions, raw p-value adjustment and export of lists of features having a significant differential expression between the conditions. All DEGs were finally mapped onto the *Nicotiana benthamiana* v1.0.1 Sol Genomics database (https://solgenomics.net/organism/Nicotianabenthamiana/genome).

### Amplification and cloning of target sequences

We amplified an approximately 100 bp long sequence of each of our target genes using specific primers (Supplementary Table 3). The Taq DNA Polymerase enzyme (5 U/μl, EnzyQuest) was employed during the Polymerase Chain Reaction (PCR) to amplify the desired target genes. The reaction also included the cDNA template, a buffer containing Mg^2+^ ions, deoxyribonucleotide triphosphates (dNTPs) and sterile water. Agarose gel electrophoresis was used to analyze the PCR results. Ethidium bromide was used as a DNA-binding dye. All amplicons were then cloned into the pGEM-T Easy vector (Promega), using the Promega pGEM-T Easy kit. The ratio of insert to plasmid was 5:1. The reaction was incubated at 25 °C for three hours before being transformed in *E. coli dh10β* competent cells.

### Real time quantitative PCR (qPCR)

A total of 1 μg of DNAseI (Roche Diagnostics)-treated RNA per biological replicate was reverse transcribed with Reverse Transcriptase (RT, Minotech Biotechnology) using deoxyribonucleotide triphosphate(s) mix (dNTPs, Invitrogen) and 250 ng of random hexamers. The reaction was incubated at 65 °C for 5 min. Following the addition of the RT buffer, 5 mm dithiothreitol (DTT) and 40 units recombinant ribonuclease inhibitor (RRI) (Takara Bio), the reaction was then incubated for 10 min at 25 °C, 60 min at 37 °C and 15 min at 72 °C. qPCR was executed in CFX CONNECT™ thermocycler (Bio-Rad Laboratories) using the Kapa SYBR Fast qPCR Kit (Kapa Biosystems), according to the kit’s datasheet. Three reference genes were used: L23, F-BOX and PP2A, based on^113^. For this experiment, four biological and three technical replicates were performed, using the following parameters: initial enzyme activation step at 95 °C for 3 min, followed by a denaturation step at 95 °C for 3 s and an annealing and extension step at 60 °C for 30 s. In all cases, 35 amplification cycles were defined, and an amplification curve was derived for each cycle. The quantification of products was associated with the exponential phase of the reaction, with the Ct value being the most important parameter (threshold cycle).

### Rights and permissions

All plant material included in this study comply with relevant institutional, national, and international guidelines and legislation. The *Nicotiana benthamiana* seeds are of the laboratory variety widely used in laboratories across the world for more than three decades now. No other plant material has been used.

### Supplementary Information

Supplementary Information 1.

Supplementary Information 2.

Supplementary Information 3.

## Data Availability

The datasets generated during and analyzed during the current study are available in the Gene Expression Omnibus (GEO) repository, GEO accession GSE230128.
