# Supplementary material for: Stress-related transcriptomic changes associated with GFP transgene expression and active transgene silencing in plants
Source: Sci Rep. 2024 Jun 10;14:13314. doi: 10.1038/s41598-024-63527-5 (PMC11164987; doi:10.1038/s41598-024-63527-5)

1- Membrane/Methylene blue

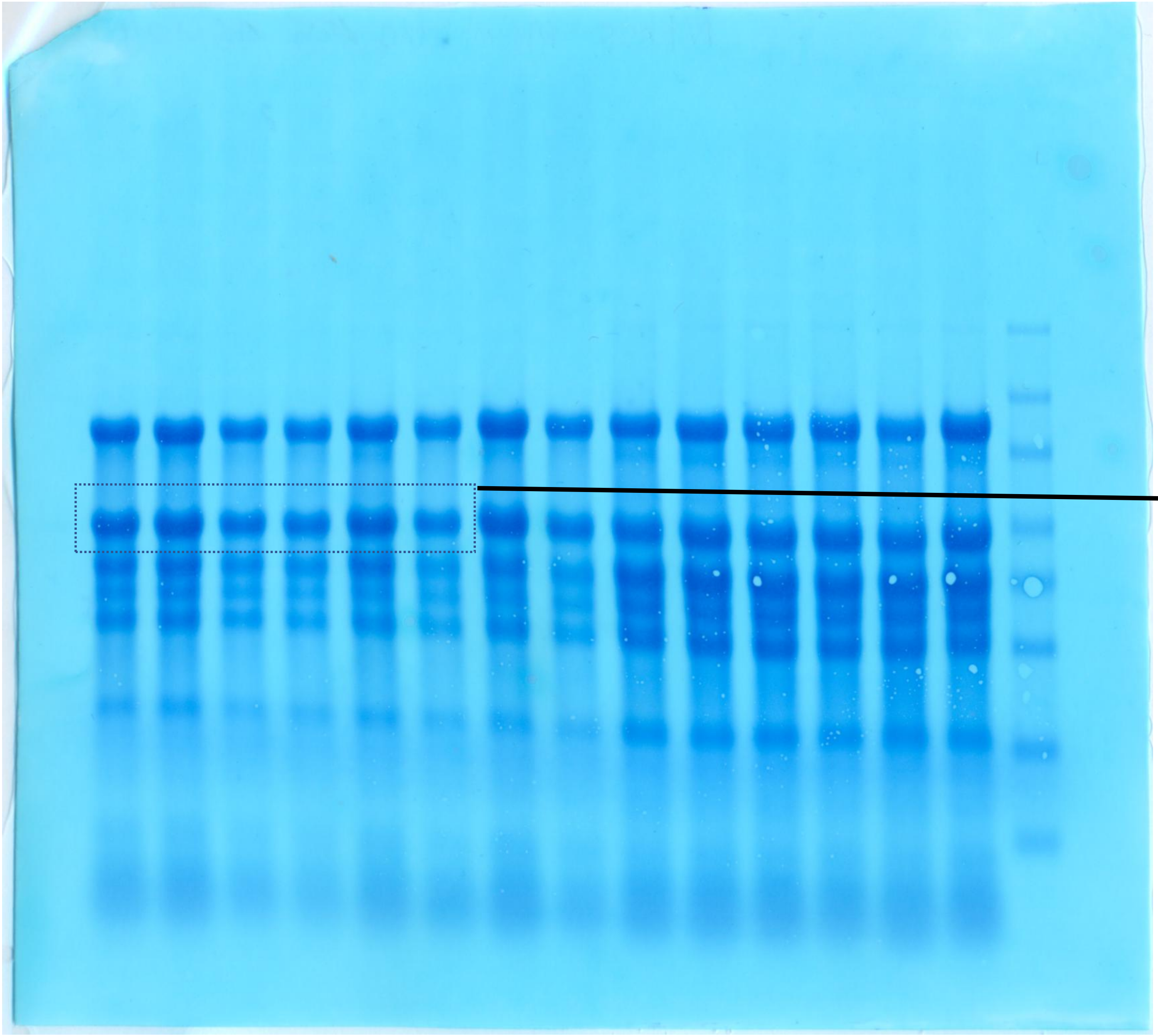

2- Film/Northern probe @mGFP (see files Film 1a and b)

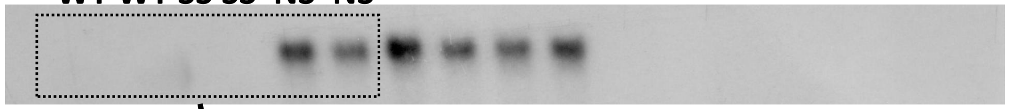

Fig.1.C

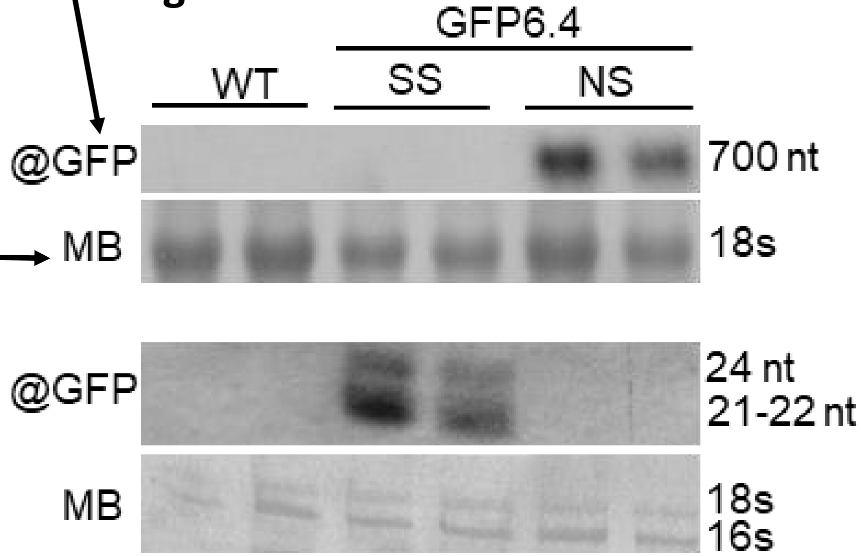

2- Film/Northern probe @mGFP (see files Film 1a and b)

Film 1a

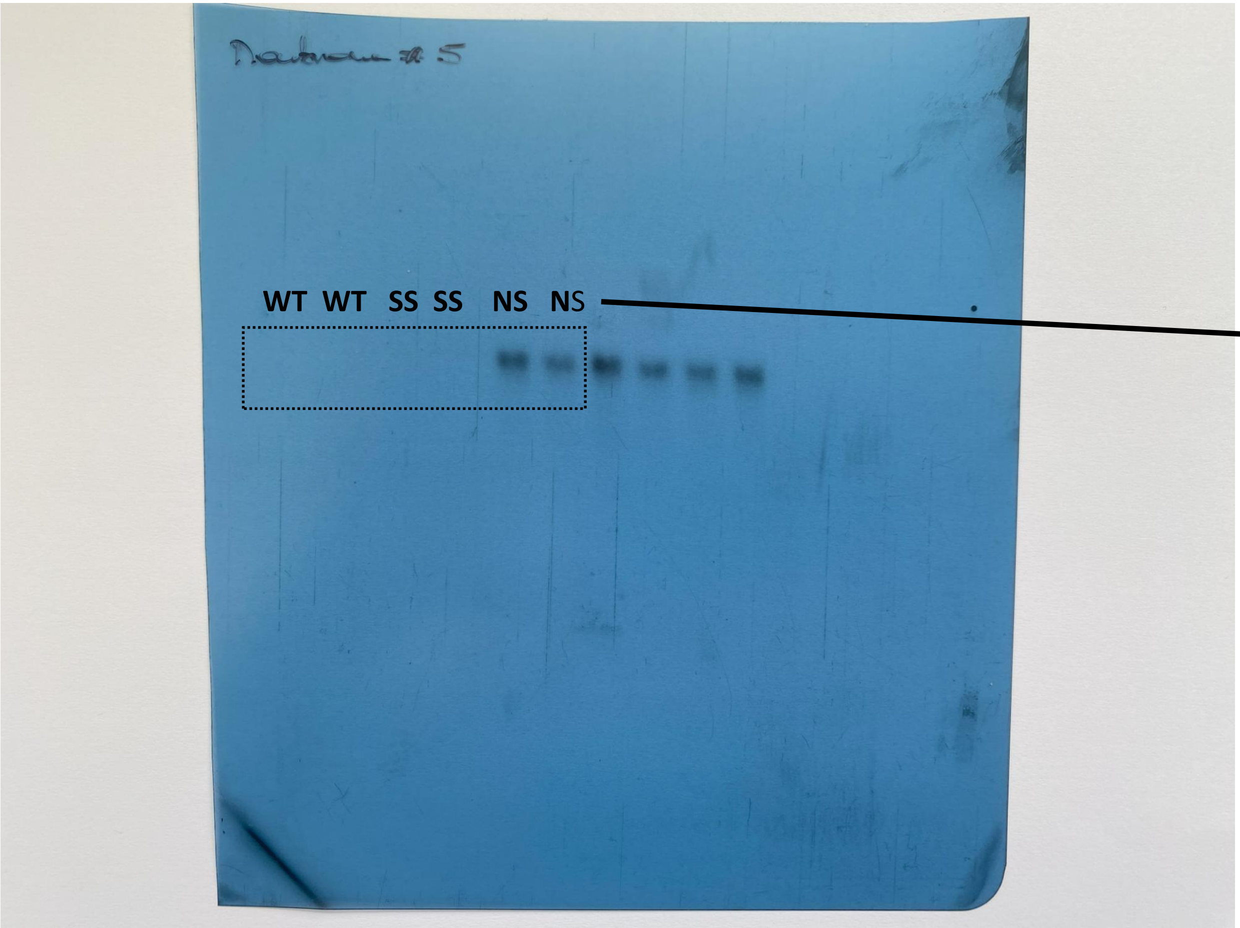

Fig.1.C

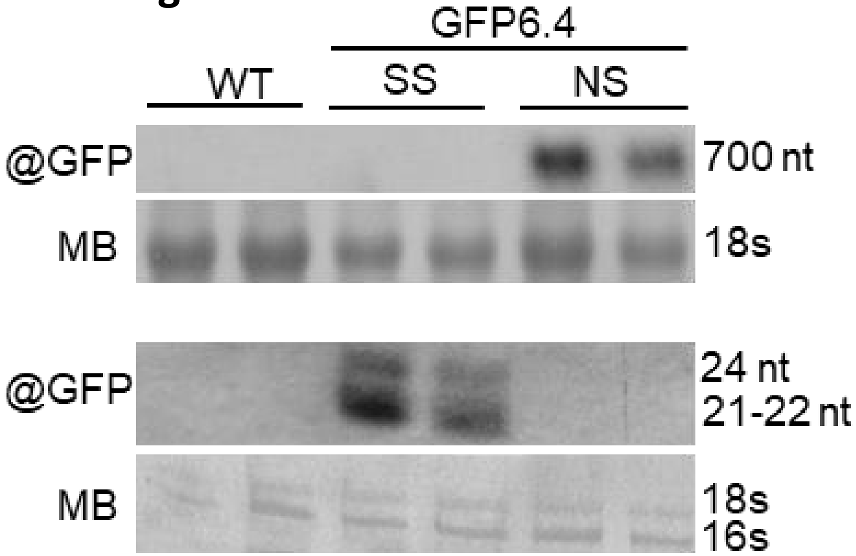

2- Film/Northern probe @mGFP (see files Film 1a and b)

Film 1b (Higher exposure of Film 1a)

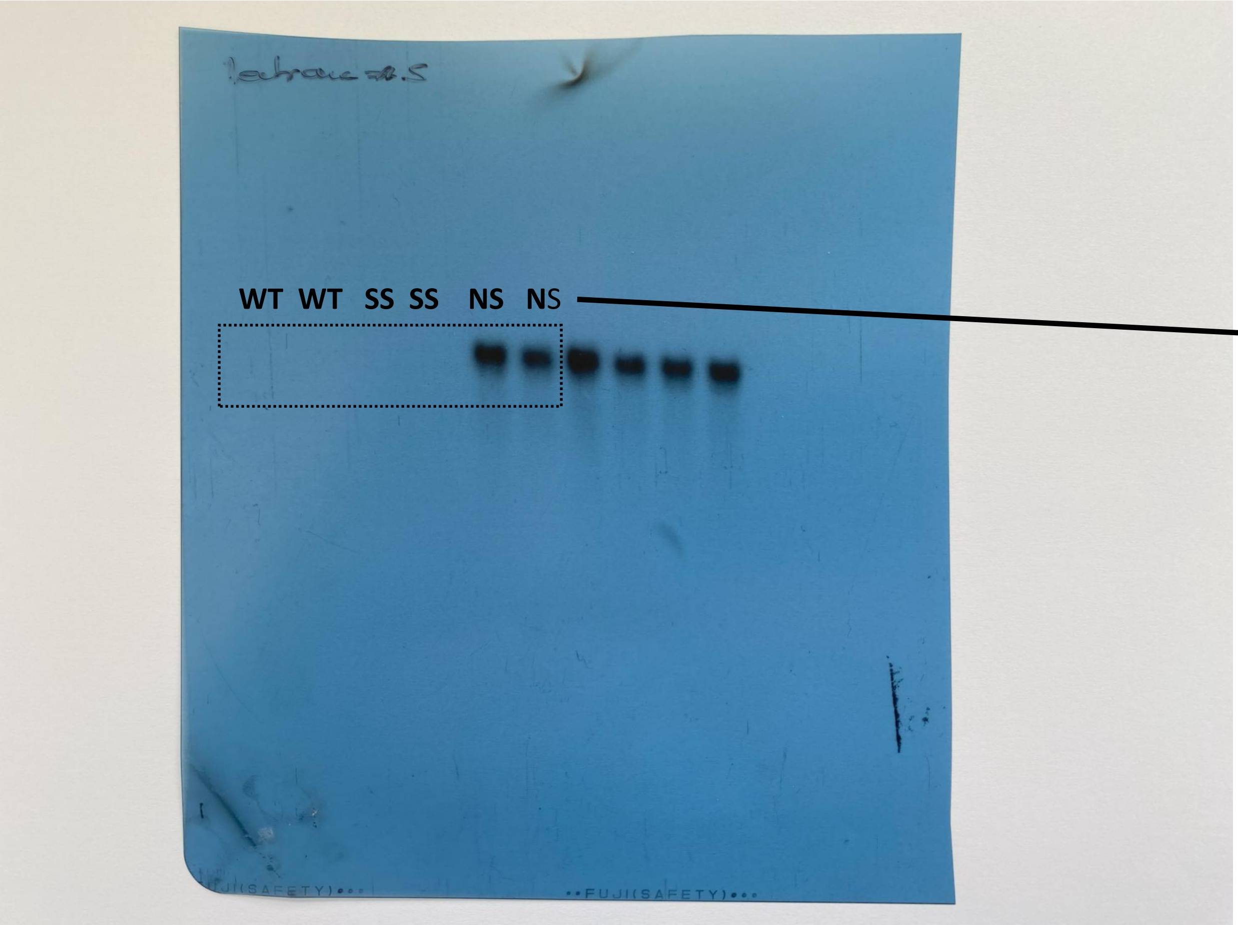

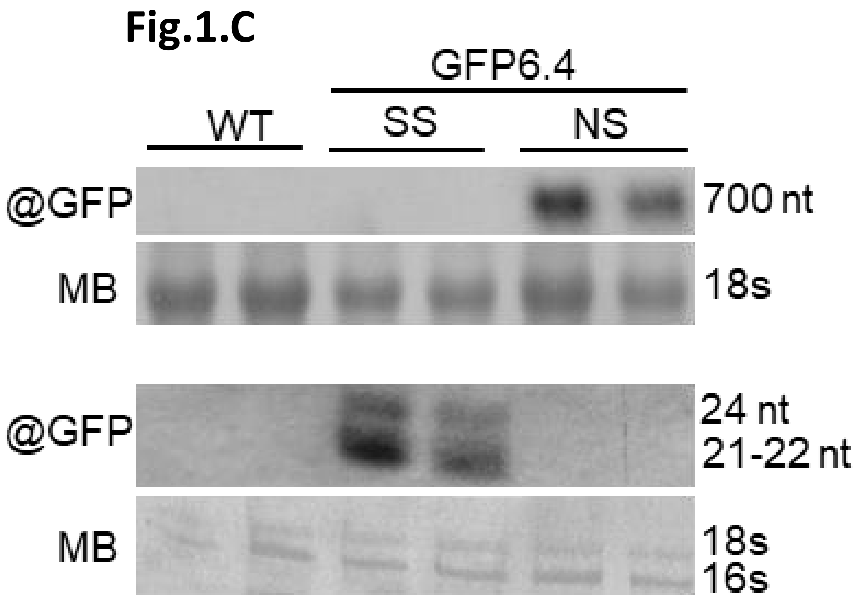

1- Membrane/Methylene blue

NS NS SS SS WT WT

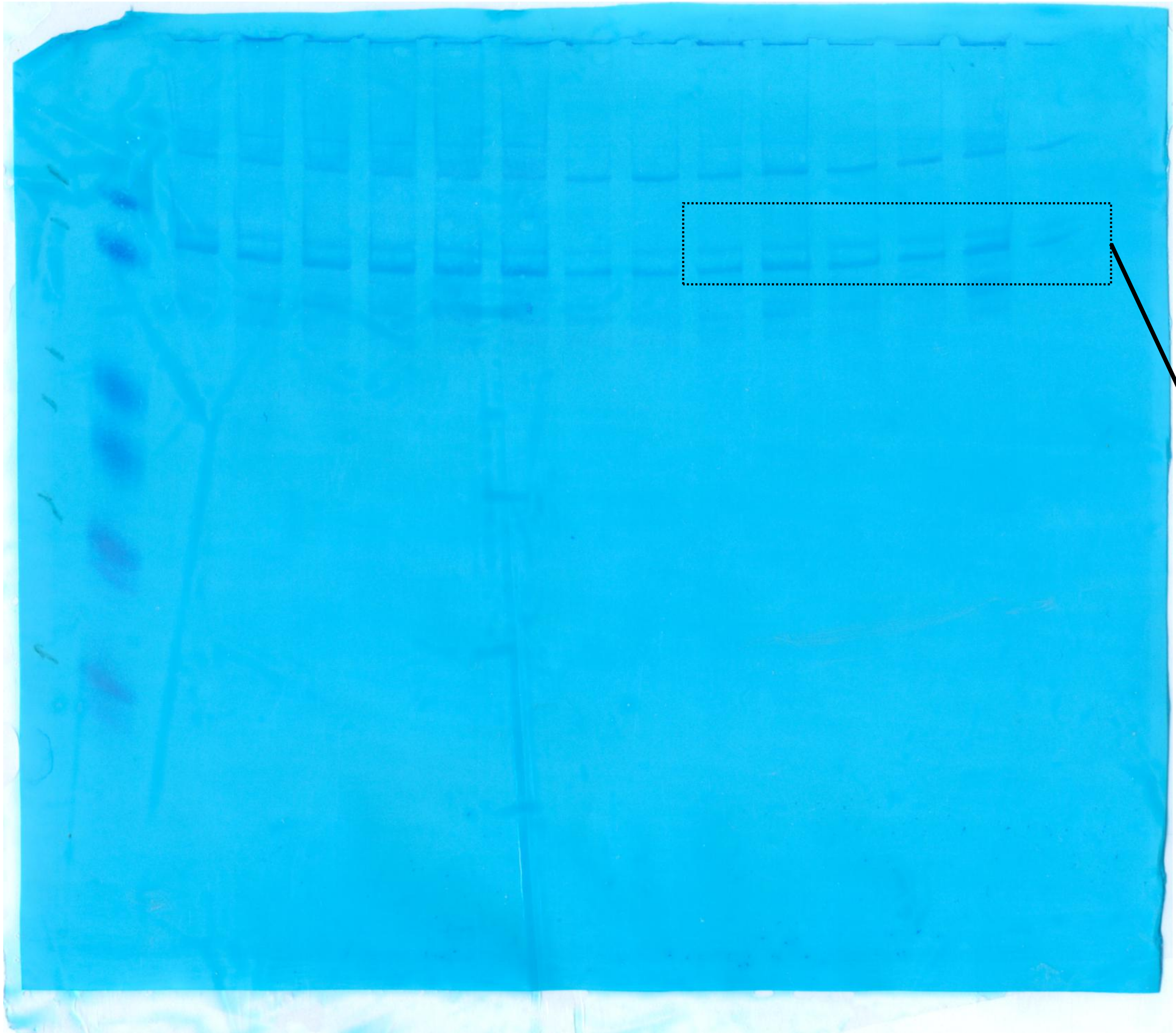

2- Film/Northern probe @mGFP (see files Film 2a and b)

NS NS SS SS WT WT

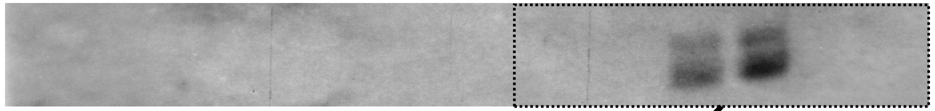

Fig.1.C

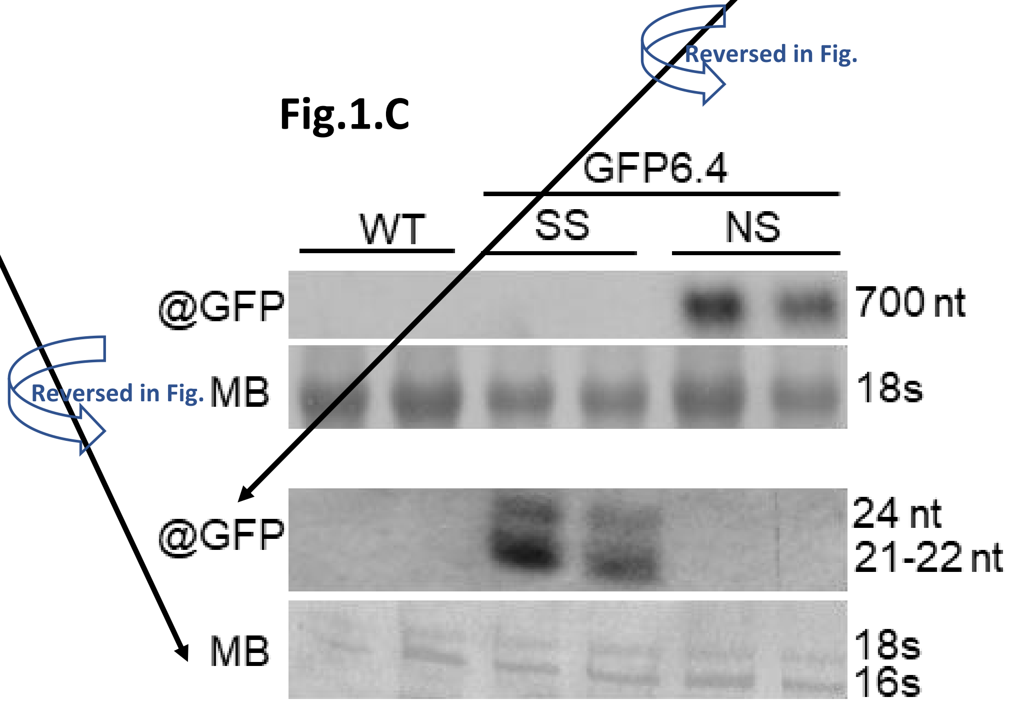

2- Film/Northern probe @mGFP (see files Film 2a and b)

Film 2a

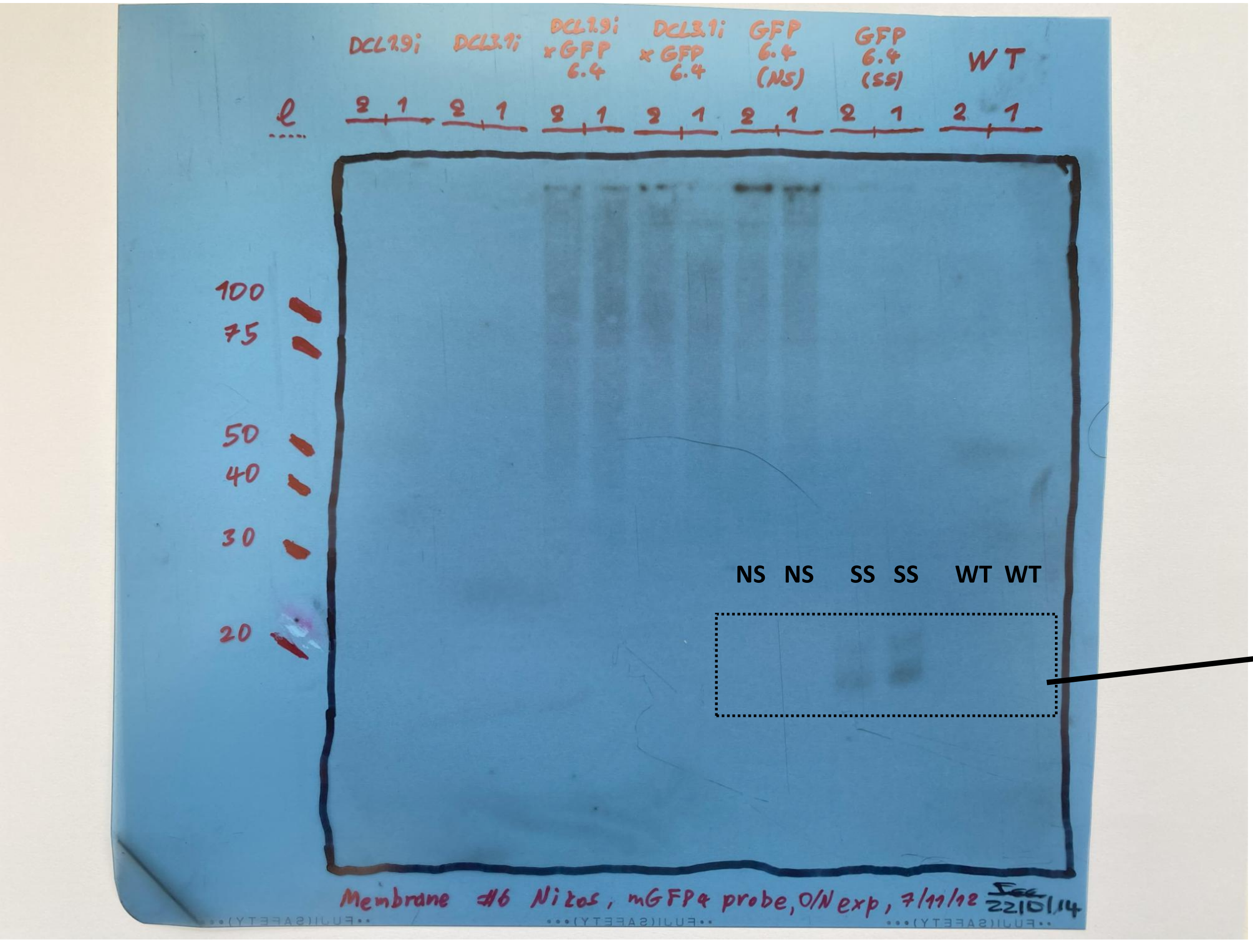

Reversed in Fig.

Fig.1.C

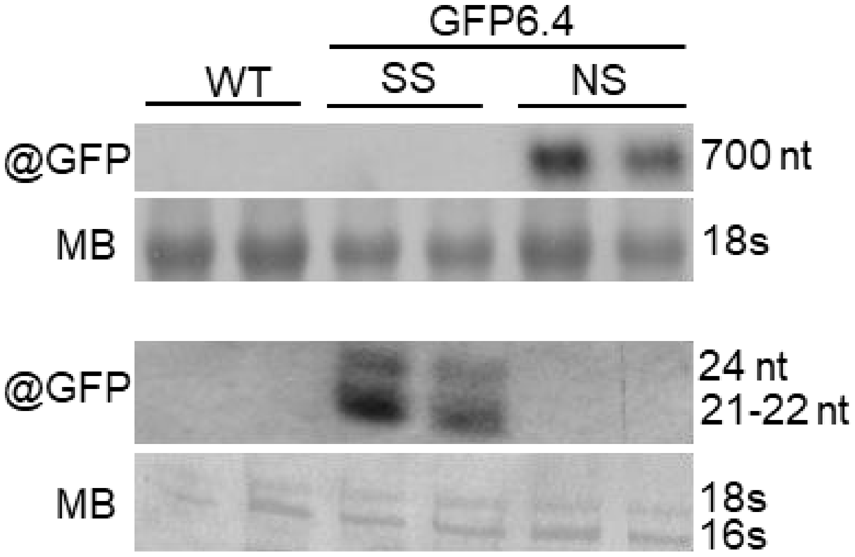

2- Film/Northern probe @mGFP (see files Film 2a and b)

Film 2b (Higher exposure of Film 2a)

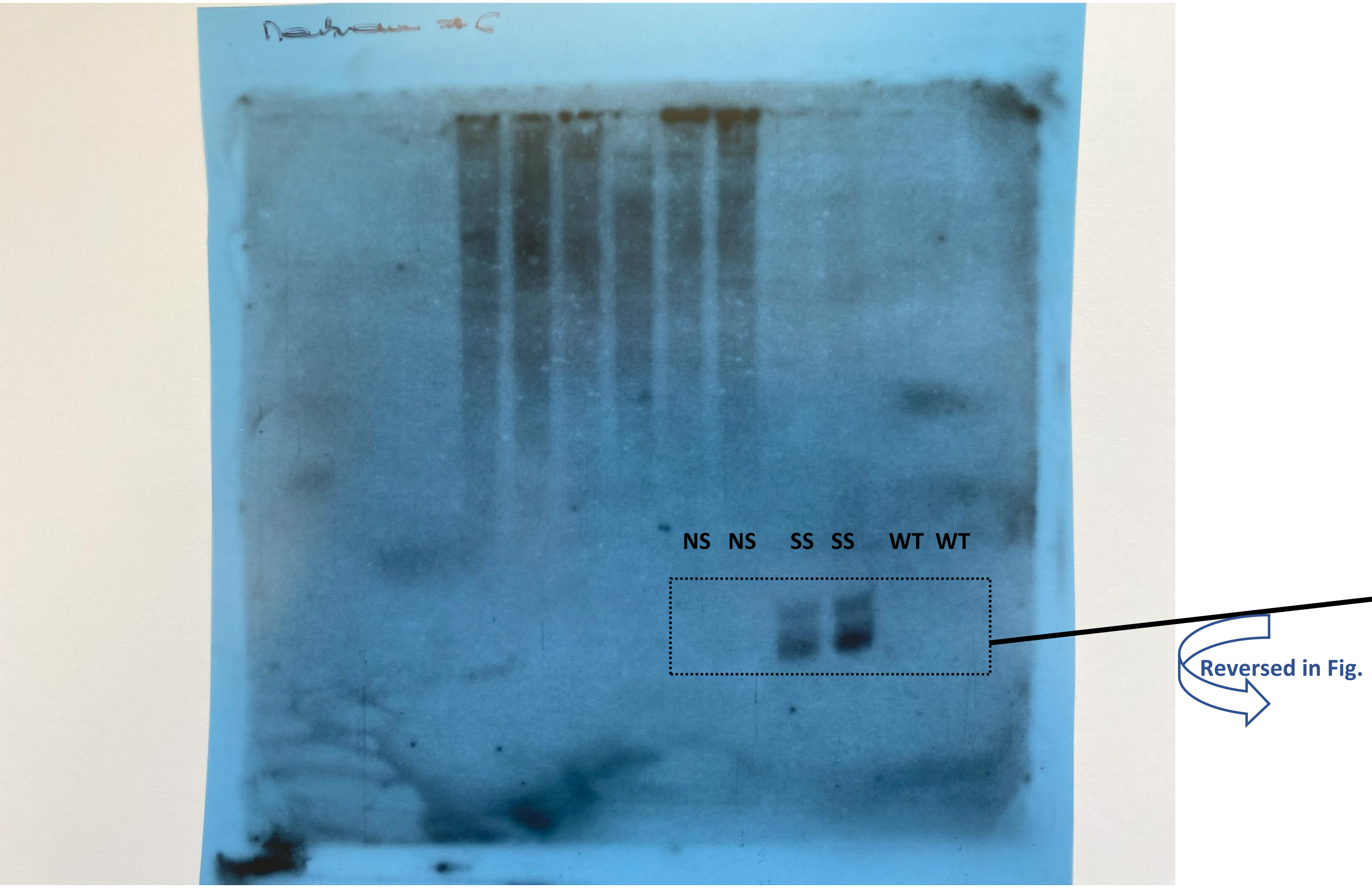

Fig.1.C

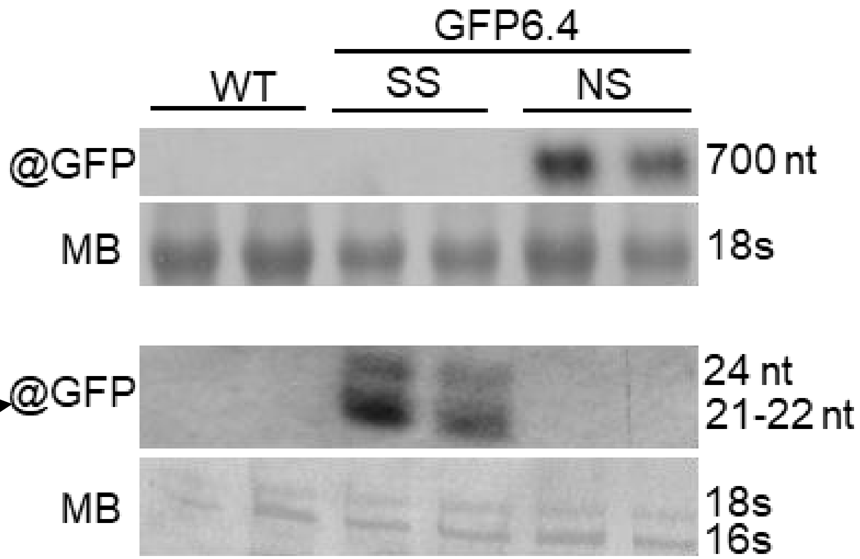

Supplement: Supplementary file 1 — Supplementary Information 1. [file 41598_2024_63527_MOESM1_ESM.pdf]
