# Supplementary material for: Stress-related transcriptomic changes associated with GFP transgene expression and active transgene silencing in plants
Source: Sci Rep. 2024 Jun 10;14:13314. doi: 10.1038/s41598-024-63527-5 (PMC11164987; doi:10.1038/s41598-024-63527-5)
Supplement: Supplementary file 3 — Supplementary Information 3. [file 41598_2024_63527_MOESM3_ESM.pdf]

Supplementary data

Supplementary Tables

Sup. Table 1 Segregation analysis of F2 progeny of five independent WTxGFP6.4 F1 plants (L1-L5) for resistance to kanamycin and GFP fluorescence. Percentage of kanamycin resistant and GFP florescent plantlets out of n seedlings germinated and grown on agar-based Murashige and Skoog medium supplemented with 100 µg.mL^{-1} kanamycin. Expected % of kanamycin resistant and GFP fluorescent plantlets for one and two unlinked T-DNAs carrying a functional nptII and mGFP4 transgenes are 75% and 93% respectively. n = number of germinated seeds.

Line	Kanamycin Resistant (%)	GFP Fluorescent (%)	n
WTxGFP6.4 L1 F2	92	92	223
WTxGFP6.4 L2 F2	92	92	90
WTxGPF6.4 L3 F2	92	92	106
WTxGFP6.4 L4 F2	93	93	60
WTxGFP6.4 L5 F2	90	90	70

Sup. Table 2 Total reads sequenced in each sample. Four biological and three technical replicates were analyzed.

Sample ID	Total number of reads (fastq)	Platform	Experiment type
**WT1**	10578188	ILLUMINA	3'Quant seq
**WT2**	9052115	ILLUMINA	3'Quant seq
**WT3**	11095121	ILLUMINA	3'Quant seq
**WT4**	10320079	ILLUMINA	3'Quant seq
**OS1**	10352753	ILLUMINA	3'Quant seq
**OS2**	11306535	ILLUMINA	3'Quant seq
**OS3**	10098292	ILLUMINA	3'Quant seq
**OS5**	10466610	ILLUMINA	3'Quant seq
**NS1**	10328154	ILLUMINA	3'Quant seq
**NS2**	9625750	ILLUMINA	3'Quant seq
**NS3**	11977619	ILLUMINA	3'Quant seq
**NS4**	9627883	ILLUMINA	3'Quant seq
**SS1**	12241420	ILLUMINA	3'Quant seq
**SS2**	10833903	ILLUMINA	3'Quant seq
**SS3**	10438408	ILLUMINA	3'Quant seq
**SS4**	10522291	ILLUMINA	3'Quant seq

Sup. Table 3 Primers for the amplification of target sequences for qPCR.

Target	Forward primer 5'- 3'		Reverse primer 5'- 3'		Tm		Amplicon size
CASP-like	CASP-F	AAGCAAACCTCCACATCTCTCA	CASP-R	CGGATATTGGAGGCAGATTACTCA	F- 58.4	R- 61.0	113bp
Peroxidase 15	PEROX-F	TGGCTCATTCTTCTTCTTTAGCCA	PEROX-R	ACTCAACTGAGCATTTGATTCATAG	F- 59.3	R- 58.1	120bp
BnaA05g03240D [Brassica napus]	BNA-F	CAGCTGGTCACCGACA	BNA-R	ACCACTGGCTTAACAGCTAAT	F- 54.3	R- 55.9	83bp
Tetratricopeptide repeat (TPR)-like superfamily	TPR-F	GAGAGAGAGGCGATGCTTTAT	TPR-R	TGATCCGAACAGCCCAATTCA	F- 57.9	R- 57.9	120bp
Cytochrome P450 superfamily	P450-F	AGATCTCCTCACTGAGTTCTGTA	P450-R	AGACACACAATAAAACTTCTTCTGG	F- 58.9	R- 58.1	107bp
C2 calcium/lipid-binding plant phosphoribosyltransferase	CAL-F	TGCCTCTGAAGTTGCTAGTCT	CAL-R	GAAGAGATGTCAGTGTTGTCGT	F- 57.9	R- 58.4	81bp
Protein kinase superfamily	KIN-F	CTCAGAAATGGGGGCTGTCA	KIN-R	TCTCCACCAAATTCTCTCCATC	F- 59.4	R- 58.4	98bp
UDP-Glycosyltransferase superfamily	UDP-F	TCCGTCGACAGGTGGATTTC	UDP-R	GGTACCCCTTGTCCAATCGC	F- 59.4	R- 61.4	105bp
Stress responsive A/B Barrel Dom	BAR-F	TGAGGAATCTTGCAATAAAGGCT	BAR-R	TGGGCCCATCTAAGAGGGTA	f- 57.1	r- 59.4	118bp
protein RESPONSE TO LOW SULFUR 1	RLS-F	TTGATCAGGCTCGGACCTAC	RLS-R	CGGTAACGGAAGCTGATTCG	F- 59.4	R- 59.4	93bp
WRKY transcription factor 44	WRKY44-F	CCCGTGTGTGAAAGGATTGC	WRKY44-R	TCCAACCTTGTTTGGAGAGGT	F- 59.4	R- 57.9	118bp

Supplementary figures

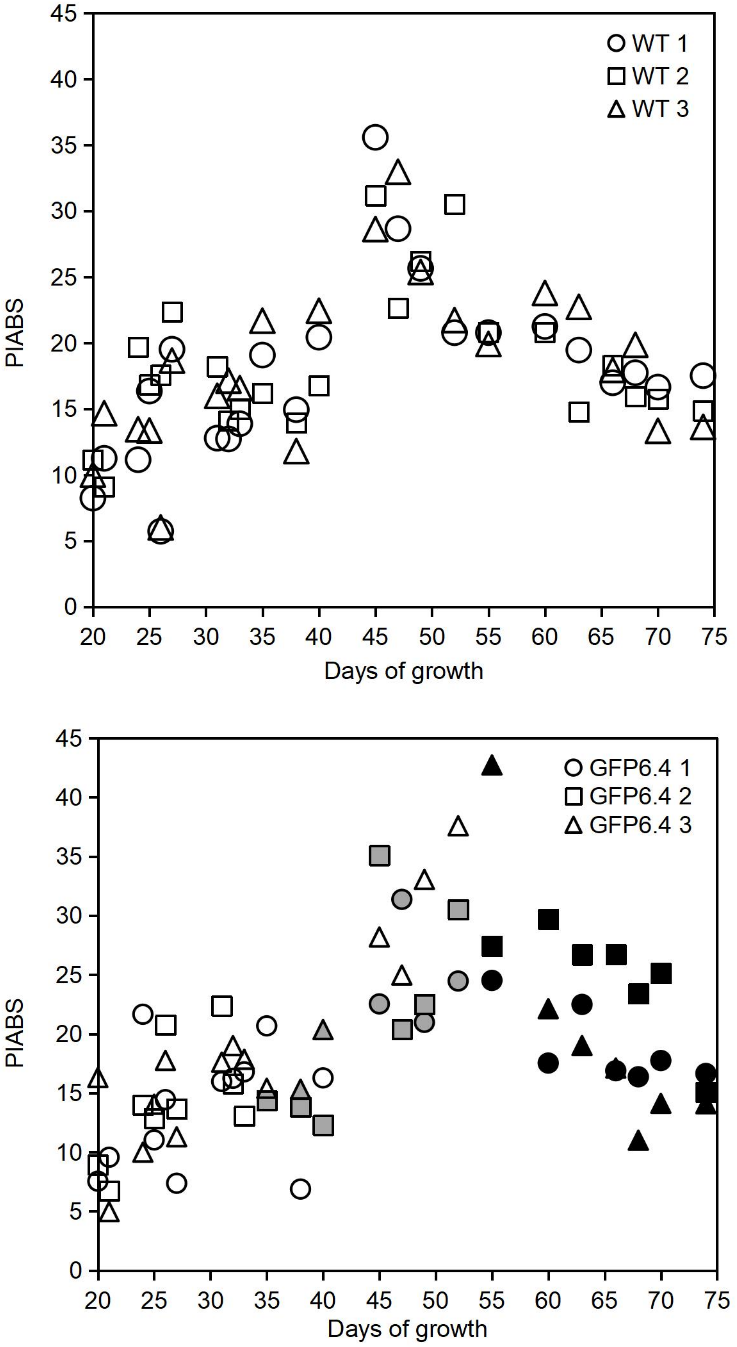

Sup. Fig. 1 Initiation and progression of GFP-PTGS do not affect photosynthetic activity in line GFP6.4. Performance index on absorption basis (PIABS) in function of time (number of days of growth) of three representative WT (a) and GFP6.4 plants (b) obtained from OJIP-test time series analysis.The OJIP test time series analysis in (b) shows the changes in the fluorescence transient over time, with white points indicating that the leaf was non-silenced in NS plants, grey points indicating that the leaf was non-silenced in plants displaying either OS or SS in distant leaf tissues,

and black points indicating that the leaf was systemically silenced in plants displaying either OS or SS in distant leaf

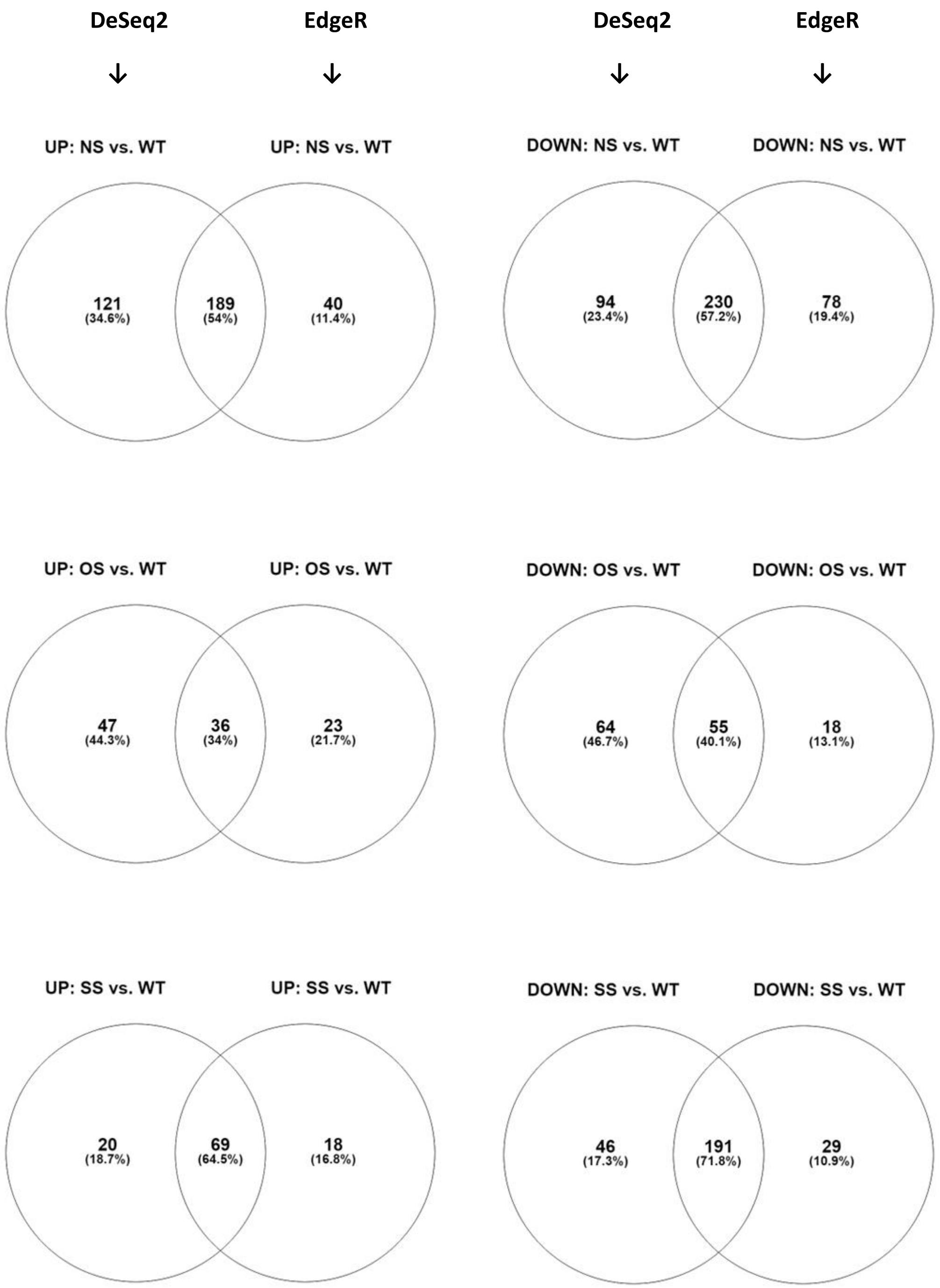

Sup. Fig.2 Transcriptional reprogramming taking place in GFP6.4 NS, OS and SS versus WT plants. Venn diagrams present up- and down-regulated gene sets.

tissues.

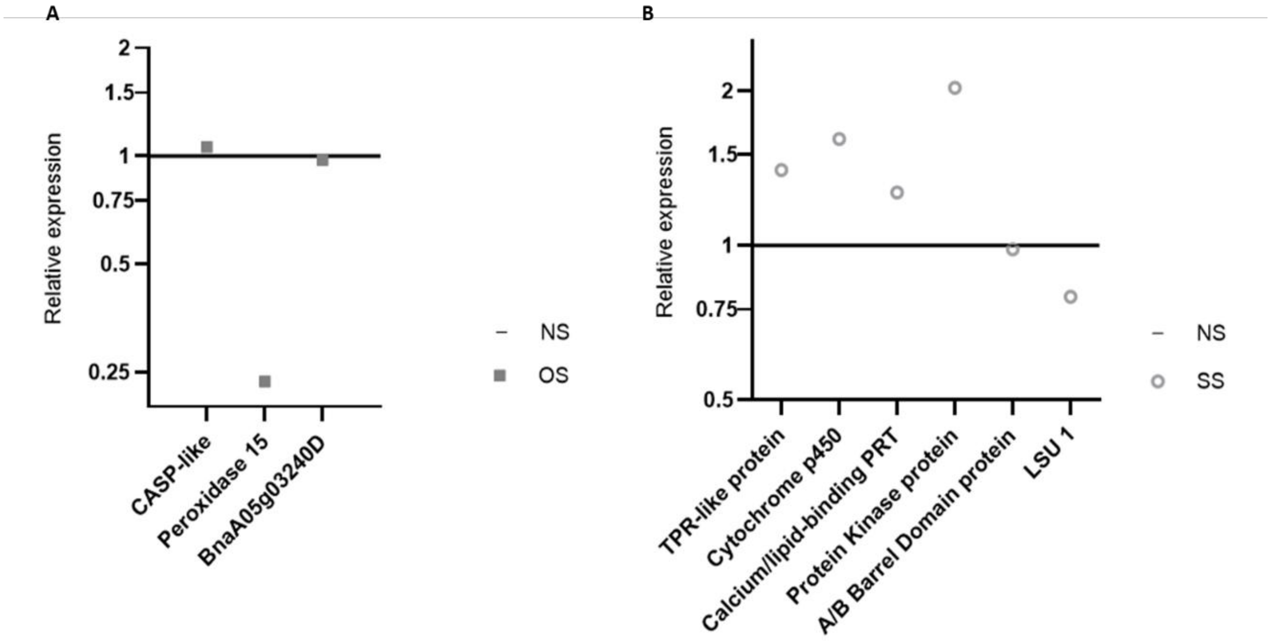

Sup. Fig.3 Differentially expressed genes between: A) NS-OS GFP6.4 plants and B) NS-SS GFP6.4 plants. qPCR quantified data are presented as relative expression levels
